# Investigating Variability in Viral Presence and Abundance across Soybean Seed Development Stages Using Transcriptome Analysis

**DOI:** 10.3390/plants12183257

**Published:** 2023-09-13

**Authors:** Hoseong Choi, Yeonhwa Jo, Hyunjung Chung, Soo Yeon Choi, Sang-Min Kim, Jin-Sung Hong, Bong Choon Lee, Won Kyong Cho

**Affiliations:** 1 Plant Health Center, Seoul National University, Seoul 08826, Republic of Korea; bioplanths@gmail.com; 2 College of Biotechnology and Bioengineering, Sungkyunkwan University, Suwon 16419, Republic of Korea; yeonhwajo@gmail.com; 3 Crop Foundation Division, National Institute of Crop Science, Rural Development Administration, Wanju 55365, Republic of Korea; chunghyunjung@korea.kr (H.C.); choisy99@korea.kr (S.Y.C.); kimsangmin@korea.kr (S.-M.K.); 4 Department of Applied Biology, Kangwon National University, Chuncheon 24341, Republic of Korea; jinsunghong@kangwon.ac.kr; 5 Crop Protection Division, National Academy of Agricultural Science, Rural Development Administration, Wanju 55365, Republic of Korea

**Keywords:** virus, viral load shifts, soybean seed development, transcriptome analysis, viromes

## Abstract

Plant transcriptomes offer a valuable resource for studying viral communities (viromes). In this study, we explore how plant transcriptome data can be applied to virome research. We analyzed 40 soybean transcriptomes across different growth stages and identified six viruses: broad bean wilt virus 2 (BBWV2), brassica yellow virus (BrYV), beet western yellow virus (BWYV), cucumber mosaic virus (CMV), milk vetch dwarf virus (MDV), and soybean mosaic virus (SMV). SMV was the predominant virus in both *Glycine max* (GM) and *Glycine soja* (GS) cultivars. Our analysis confirmed its abundance in both, while BBWV2 and CMV were more prevalent in GS than GM. The viral proportions varied across developmental stages, peaking in open flowers. Comparing viral abundance measured by viral reads and fragments per kilobase of transcript per million (FPKM) values revealed insights. SMV showed similar FPKM values in GM and GS, but BBWV2 and CMV displayed higher FPKM proportions in GS. Notably, the differences in viral abundance between GM and GS were generally insignificant based on the FPKM values across developmental stages, except for the apical bud stage in four GM cultivars. We also detected MDV, a multi-segmented virus, in two GM samples, with variable proportions of its segments. In conclusion, our study demonstrates the potential of plant transcriptomes for virome research, highlighting their strengths and limitations.

## 1. Introduction

Soybean (*Glycine max* L.) is one of the most important crops globally, providing a vital source of protein and oil for human consumption, livestock feed, and industrial applications [1]. As soybean production expands across diverse regions, the threat of viral diseases poses a significant challenge to sustainable cultivation and global food security [2].

The soybean virome, encompassing the collective viral population associated with soybean plants, plays a critical role in shaping disease dynamics and crop productivity [3]. Viral pathogens can cause a wide range of symptoms in soybean, including mosaic patterns, leaf distortion, stunting, and reduced seed quality [4]. Among the numerous viruses affecting soybean, several have gained prominence due to their economic impact and widespread distribution.

Soybean mosaic virus (SMV) is a single-stranded positive-sense RNA virus belonging to the genus *Potyvirus* in the family *Potyviridae* and is regarded as one of the most significant viral pathogens that impacts soybean on a global scale [5,6]. SMV infects a wide range of soybean cultivars and wild relatives, leading to severe yield losses and significant reductions in seed quality [7]. The virus is primarily transmitted through aphids and can also be seed-borne, further contributing to its dissemination and persistence within soybean populations [8].

In addition to SMV, several other RNA viruses have been identified in soybean, including soybean vein necrosis-associated virus (SVNaV), bean pod mottle virus (BPMV), soybean yellow mottle mosaic virus (SYMMV), soybean yellow common mosaic virus (SYCMV), peanut stunt virus (PSV), peanut mottle virus (PeMoV), and alfalfa mosaic virus (AMV) [2,3]. Furthermore, recent studies have uncovered the presence of novel geminiviruses infecting soybean; however, the disease symptoms caused by these geminiviruses in soybean plants are largely asymptomatic [9,10]. These viruses, along with their variants and strains, exhibit diverse geographic distributions and host ranges, highlighting the global nature of the soybean virome.

Recent advances in high-throughput RNA sequencing (RNA-seq) technologies have revolutionized the field of virology, enabling comprehensive characterization of viral communities within different host organisms [11]. Transcriptome analysis using RNA-seq provides valuable insights into the expression patterns and interactions between host genes and viral genomes [12]. By applying RNA-seq to the investigation of the soybean virome during seed development, we can gain a deeper understanding of viral diversity, prevalence, and dynamics throughout this critical developmental stage.

Understanding the dynamics of viral infections during soybean seed development is crucial for devising effective management strategies. The seed stage is a pivotal phase in plant development, and it presents a potential avenue for the transmission of viral pathogens [13]. Therefore, unraveling the soybean virome during seed development is essential for comprehending the viral ecology and identifying key factors influencing the viral prevalence and load.

The RNA-seq technique has been utilized for various purposes in plant sciences, including gene expression profiling, transcriptome assembly, and annotation [14]. Many transcriptome-related studies employing RNA-seq utilize libraries with poly(A) tails to assess the expression of messenger RNAs. Despite researchers’ efforts to control for environmental factors in plant samples, these samples are often infected by different viruses that escape detection by plant biologists. While ribosome depletion libraries are commonly employed for plant virome studies, mRNA libraries employing oligo-d(T) priming can contain a substantial number of viruses and viroids [15,16]. Nevertheless, not all plant transcriptomes are conducive to virome studies due to multiple unidentified factors. 

In this study, we leveraged previous RNA-seq data to explore the viromes associated with cultivated and wild soybean varieties, namely *Glycine max* L. and *Glycine soja* L., respectively, throughout soybean seed development [17]. By amassing transcriptome data from diverse cultivars across various developmental stages, our goal was to comprehensively identify prevalent viruses and scrutinize their fluctuations in viral load. Furthermore, we deliberated on the potential application of plant transcriptomes for virome studies, delineating their advantages and limitations.

## 2. Results

### 2.1. Identification of Viruses from 40 Soybean Transcriptome Datasets

**Figure 1:**
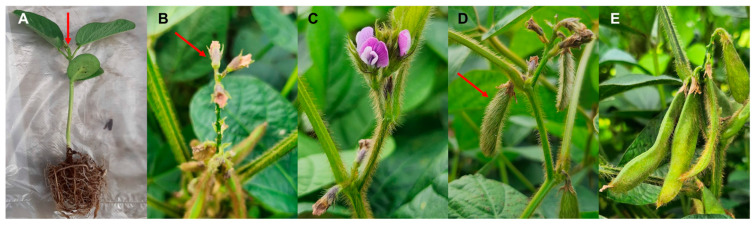
Images of soybean tissues at five developmental stages. All images were captured by Won Kyong Cho in Gadam-ri, Hoengseong-gun, Republic of Korea. (**A**) Apical bud, (**B**) flower bud, (**C**) open flower, (**D**) developing pod at five days post-fertilization, (**E**) developing pod at fifteen days post-fertilization. Red-colored arrows are used to highlight each specific tissue.

We analyzed data from a previous transcriptome study that included four different cultivars of *Glycine max* (GM) and *Glycine soja* (GS) (Table 1) [17]. These soybean cultivars exhibited variations in flower and seed coat color. RNA sequencing was performed on samples collected at five distinct developmental stages: apical buds (ABs), flower buds (FBs), open flowers (OFs), and developing pods at five (P05) and 15 days (P15) post-fertilization (Figure 1). Individual soybean cultivar names, such as GM1 to GM4 and GS1 to GS4, were abbreviated for simplicity (Table 1).

**Figure 2:**
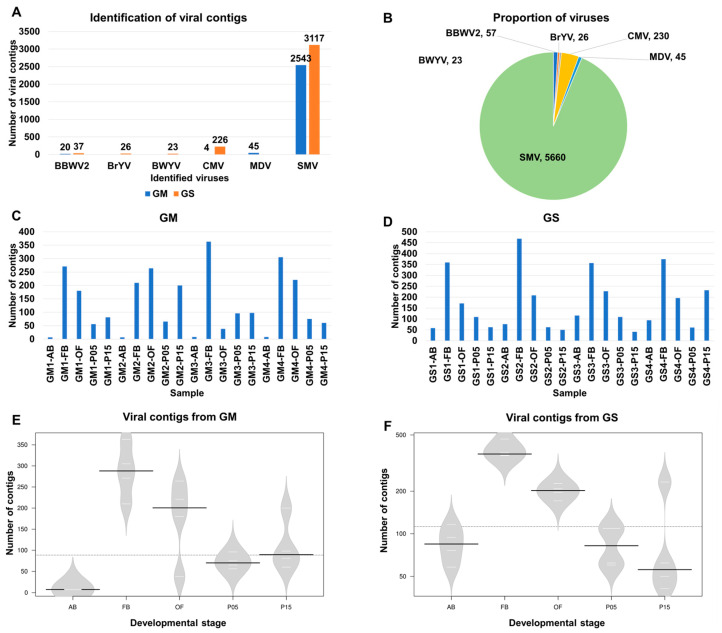
The identification of viruses from 40 transcriptomes’ data derived from *Glycine max* (GM) and *Glycine soja* (GS). (**A**) The number of viral contigs for the identified viruses from GM and GS are labeled in blue and orange, respectively. (**B**) The proportion of identified viruses is shown by combining 40 soybean transcriptomes based on the number of contigs assigned to each virus. The number of viral contigs in each sample derived from GM (**C**) and GS (**D**). Bean plots display the number of viral contigs in each developmental stage for GM (**E**) and GS (**F**). In the bean plot, the black solid lines represent the median values, while the white lines depict individual data points.

The raw data from each library underwent trimming procedures and are summarized in Table S1. Following the trimming phase, the resulting clean reads were subjected to de novo assembly utilizing the Trinity program. Subsequently, these assembled sequences were subjected to BLASTX (version 2.12.0.) searches against a viral protein database. Consequently, a total of 2613 virus-associated contigs (referred to as viral contigs) were identified from GM, while 3429 viral contigs were identified from GS. These contigs were attributed to six different viruses: broad bean wilt virus 2 (BBWV2), brassica yellow virus (BrYV), beet western yellow virus (BWYV), cucumber mosaic virus (CMV), milk vetch dwarf virus (MDV), and SMV (Table S2). Among these, SMV accounted for the highest abundance, with 2543 and 3117 contigs detected in GM and GS, respectively. CMV followed as the second-most-abundant virus, with 4 and 226 contigs identified in GM and GS, respectively. BBWV2 ranked third, with 20 and 37 contigs found in GM and GS, respectively (Figure 2A). Additionally, CNSV (1 contig) and MDV (45 contigs) were exclusively detected in GM, while BrYV (26 contigs) and BWYV (23 contigs) were solely identified in GS (Figure 2B). Based on the number of viral contigs, SMV represented the most prevalent virus, with 5660 contigs, followed by CMV (230 contigs), BBWV2 (57 contigs), and BrYV (26 contigs).

Next, we examined the distribution of viral contigs among the different samples (Figure 2C,D). Among the 20 samples collected from GM, three samples obtained from FB, namely GM3-FB (364 contigs), GM4-FB (305 contigs), and GM1-FB (271 contigs), exhibited a higher number of viral contigs (Figure 2C). Conversely, samples collected from AB, such as GM1-AB (seven contigs), GM2-AB (seven contigs), GM3-AB (eight contigs), and GM4-AB (eight contigs), had a lower number of viral contigs. As for GS, GS2-FB (468 contigs), GS4-FB (374 contigs), GS1-FB (359 contigs), and GS3-FB (357 contigs) exhibited higher abundances of viral contigs, while GS3-P15 (41 contigs), GS2-P15 (50 contigs), and GS1-AB (58 contigs) exhibited lower numbers (Figure 2D).

Furthermore, we analyzed the number of viral contigs across the five developmental stages using violin plots (Figure 2E,F). Both in GM and GS, FB exhibited the highest number of viral contigs, followed by OF. In GM, AB displayed a lower number of viral contigs, while P15 in GS showed the lowest number. Welch’s two-sample t-tests revealed a significant difference in the number of viral contigs between FB and AB in both GM (*p* = 0.003149) and GS (*p* = 0.0003477).

### 2.2. Analysis of Viral Abundance Using Viral Reads

While viral contigs are valuable for virus identification and obtaining complete viral genomes, it is important to note that the number of viral contigs alone does not directly indicate the abundance of the identified virus in each sample. To ensure a precise evaluation of viral abundance, our analysis was centered on viral reads (Table S3). Furthermore, we computed the coverage for individual virus segments within each sample (Table S4).

**Figure 3:**
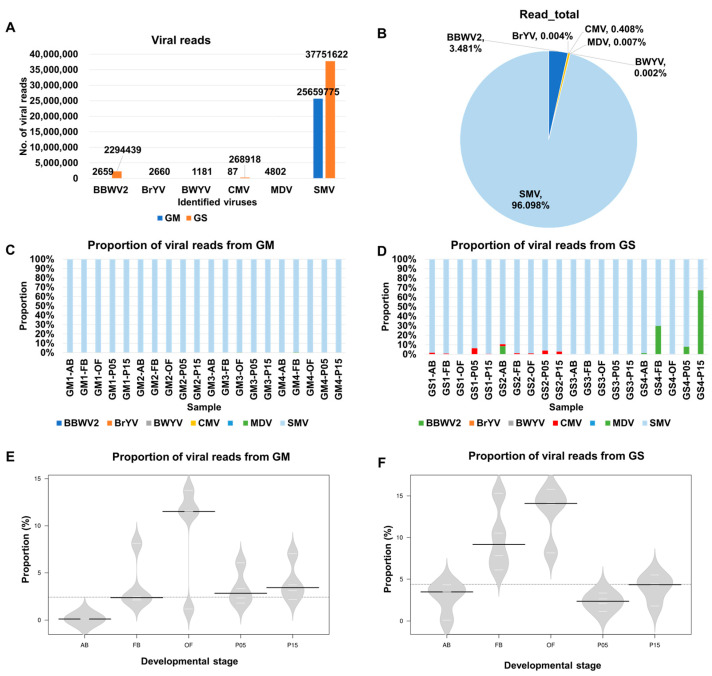
The number of viral reads assigned to each virus and the proportion of viral reads in each sample. (**A**) The number of viral reads for the identified viruses is shown by combining 40 soybean transcriptomes, with blue and orange colors indicating GM and GS, respectively. (**B**) The proportion of viruses is shown according to the viral reads assigned to each virus. The proportion of viral reads in each sample derived from GM (**C**) and GS (**D**) is displayed. The proportion of viral reads in each developmental stage derived from GM (**E**) and GS (**F**) is shown. In the bean plot, the black solid lines represent the median values, while the white lines depict individual data points.

Based on viral reads, SMV exhibited the highest abundance, with 25,659,775 and 37,751,622 reads from GM and GS, respectively. Following SMV, BBWV2 had 2659 and 2,294,439 reads from GM and GS, respectively, while CMV had 87 and 268,918 reads from GM and GS, respectively (Figure 3A). When considering all viral reads combined from the 40 samples, SMV accounted for the majority, with 96.1%, followed by BBWV2, with 3.48% (Figure 3B). The proportions of the other five viruses, namely CMV, BrYV, BWYV, CNSV, and MDV, were all less than 1% (Figure 3B).

We examined the distribution of the identified viruses based on viral reads in each transcriptome (Figure 3C,D). Interestingly, in all 20 samples from GM, SMV constituted the majority of viral reads, while the proportions of the other viruses were significantly lower (Figure 3C). In the case of GS, SMV remained the most abundant virus in most samples, except for a few instances, such as GS4-P15, where BBWV2 accounted for 67.1% of the viral reads (Figure 3D). CMV was detected in 13 samples, yet its highest proportion reached 6.2%, notably in GS1-P15. BBWV2 was identified in 10 samples, and 4 of them (GS2-AB, GS4-FB, GS4-P05, and GS4-P15) displayed a higher proportion of BBWV2.

Furthermore, we examined the proportions of viral reads in individual transcriptomes according to the five developmental stages (Figure 3E,F). Open flowers (OFs) exhibited the highest proportion of viral reads, averaging 9.5% for GM and 13% for GS. Following OFs, flower buds (FBs) had 3.8% viral reads for GM and 9.9% for GS. In GM, the viral proportions were generally low, ranging from 0% to 0.1%, and the average viral proportions for FBs (3.8%), P05 (3.4%), and P15 (4%) were relatively similar (Figure 3E). In GS, the viral proportions for FBs (9.9%) and OFs (13%) were significantly higher than those of the other three developmental stages: ABs (3.0%), P05 (2.3%), and P15 (4.0%) (Figure 3F).

### 2.3. Analysis of Viral Abundance Using Fragments per Kilobase of Transcript per Million (FPKM) Values

The viral read count represents the number of reads that are mapped to the viral genome. However, the fragments per kilobase of transcript per million (FPKM) values take into account sequencing depth and feature length when calculating gene expression levels. It is important to note that the choice of computational methods for gene expression analysis using RNA sequencing data can impact the estimation of viral abundance in each transcriptome [18]. To assess the differences in viral abundance between viral reads and FPKM values, we calculated the FPKM values for the identified viruses in each transcriptome (Table S5).

**Figure 4:**
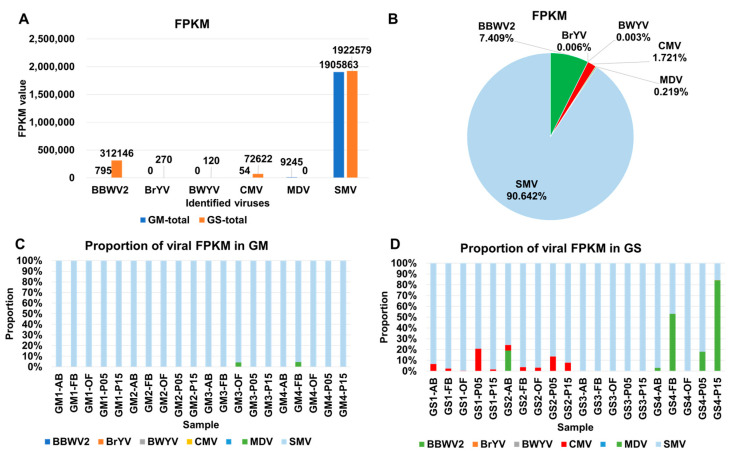
The proportion of identified viruses in each sample is based on the FPKM (fragments per kilobase of transcript per million) value. (**A**) The FPKM value representing viral transcripts for each virus is shown, with blue and orange colors indicating GM and GS, respectively. (**B**) The proportion of identified viruses is shown by combining 40 soybean transcriptomes based on the FPKM values. The proportion of viral transcripts in each sample based on the FPKM values is displayed for GM (**C**) and GS (**D**).

Based on the number of viral contigs and viral reads, there was a significant difference in SMV abundance between GM and GS. However, based on FPKM values, the viral abundance of SMV was quite similar between GM (1,905,863) and GS (1,922,579) (Figure 4A). The viral abundance of BBWV2 and CMV was much higher in GS compared with GM. When combining all FPKM values from the 40 transcriptomes, SMV accounted for 90.64%, followed by BBWV2 (7.41%) and CMV (1.72%) (Figure 4B). The proportions of the other four viruses were, again, less than 1%.

In each sample, SMV was the predominant virus based on the FPKM values in GM. However, the proportion of MDV in samples GM3-OF and GM4-FB was 4.3%, which is much higher than that obtained from viral reads (Figure 4C). Similarly, the proportions of BBWV2 and CMV were increased in GS based on the FPKM values compared with viral reads (Figure 4D). For example, the proportion of CMV was very high in GS1-AB (6.6%), GS1-P05 (20.7%), GS2-P05 (13.4%), and GS2-P15 (7.8%), whereas the proportion of BBWV2 was very high in GS2-AB (19.1%), GS4-FB (53%), GS4-P05 (17.9%), and GS4-P15 (84%) (Figure 4D).

### 2.4. Comparison of Viral Abundance Based on FPKM Values

**Figure 5:**
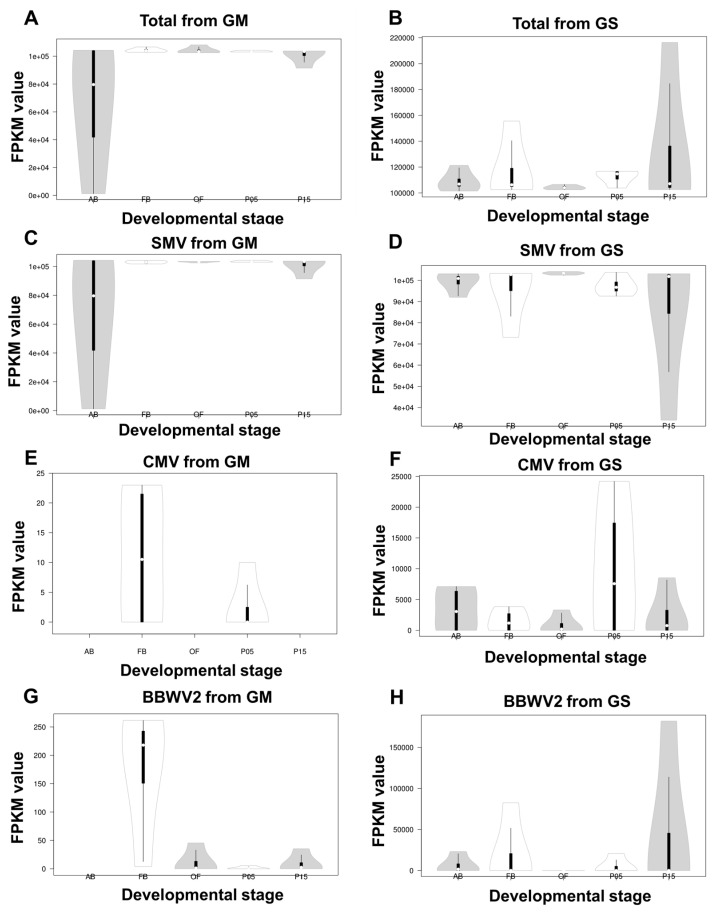
The proportion of viral transcripts in each developmental stage based on the FPKM values. Violin plots display the distribution of the total amount of viral transcripts derived from four samples for each soybean developmental stage for GM (**A**) and GS (**B**). The distribution of viral transcripts in each soybean developmental stage for SMV is shown for both GM (**C**) and GS (**D**). The distribution of viral transcripts in each soybean developmental stage for CMV is shown for both GM (**E**) and GS (**F**). The distribution of viral transcripts in each soybean developmental stage for BBWV2 is shown for both GM (**G**) and GS (**H**).

We conducted a comparison of viral abundance between GM and GS at five different developmental stages based on the FPKM values (Figure 5A,B). No significant differences were observed in viral abundance among the five developmental stages in both GM and GS, as indicated by the FPKM values. However, we did find a significant difference in viral abundance among the four different GM cultivars at the AB stage. GM1 (FPKM = 104,297) and GM4 (FPKM = 103,946) exhibited higher viral abundance compared with GM2 (FPKM = 55,340) and GM3 (FPKM = 1231) (Figure 5A).

Furthermore, we compared viral abundance between GM and GS at the five different stages using t-tests. The resulting *p*-values were 0.17781827 (ABs), 0.37051455 (FBs), 0.94962009 (OFs), 0.05911304 (P05), and 0.32243160 (P15), all of which were greater than 0.05, indicating no significant difference between the two (Figure 5B). 

Regarding the abundance of SMV, there was a high abundance of SMV among different GM cultivars at the AB stage (Figure 5C), while among different GS cultivars, SMV abundance was notably high at the P15 stage (Figure 5D). Once again, the t-test *p*-values for SMV abundance were all greater than 0.05, indicating no significant difference between the cultivars at any of the five stages. 

In the case of CMV, its abundance was considerably lower in GM compared with GS (Figure 5E,F). Moreover, CMV was only identified in two stages in GM, while it was identified in all five stages in GS. BBWV2, on the other hand, was identified in four stages in GM, but its abundance was lower compared with that in GS (Figure 5G,H). In GS, BBWV2 was identified in all five stages, and the P15 stage exhibited higher variance among different GS cultivars.

### 2.5. Viral Abundance of Viruses Composed of Multiple Segments

**Figure 6:**
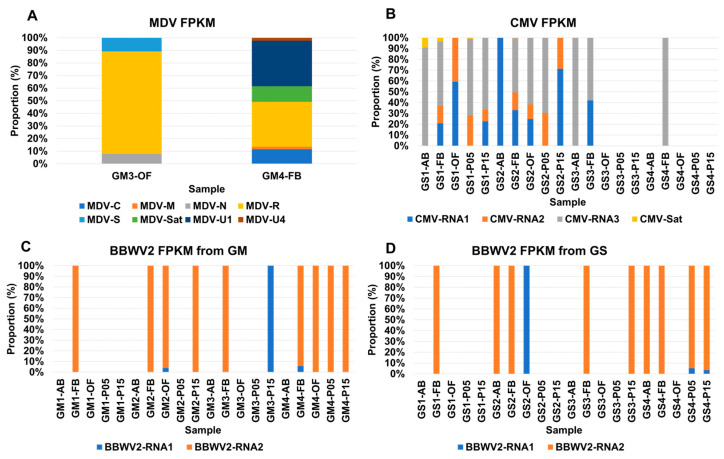
The comparison of viral transcripts for viruses composed of multiple genome segments. (**A**) A comparison of viral transcripts among DNA segments of MDV in two different samples. (**B**) A comparison of viral transcripts among RNA segments of CMV in different samples. (**C**) A comparison of viral transcripts between two RNA segments of BBWV2 in different samples from GM. (**D**) A comparison of viral transcripts between two RNA segments of BBWV2 in different samples from GS.

Some viruses, such as MDV, consist of multiple segments. In this study, MDV was identified in two samples, GM3-OF and GM4-FB from GM. We were able to identify seven segments of MDV, along with its satellite DNA. The proportion of individual segments of MDV in the two samples, based on the FPKM values, is depicted in a stacked bar graph (Figure 6A). In both samples, the MDV R segment exhibited the highest proportion, accounting for 81.2% in GM3-OF and 35.6% in GM4-FB. In GM3-OF, only three DNA segments, namely N, R, and S, were identified. On the other hand, GM4-FB exhibited six DNA segments, including C, M, N, R, U1, and U4, and the satellite DNA. The viral abundance of the R and U1 segments was quite similar in GM4-FB.

Similarly, CMV is composed of three RNA segments and a single satellite RNA. In this study, CMV was identified in 13 out of 20 GS samples (Figure 6B). The presence of each CMV RNA segment varied across the samples: CMV RNA1 was found in 8 samples, RNA2 in 8 samples, RNA3 in 10 samples, and the satellite RNA in 2 samples. Four samples, namely GS1-FB, GS1-P15, GS2-FB, and GS2-OF, contained all three CMV RNA segments, with RNA3 being the most abundant, followed by RNA1 and RNA2. 

On the other hand, BBWV2 consisted of two RNA segments, RNA1 and RNA2. Interestingly, BBWV2 was identified in 10 samples from both GM and GS (Figure 6C,D). Among the 10 GM samples infected by BBWV2, only BBWV2 RNA2 was identified in 9 samples, while RNA1 of BBWV2 was found in a single sample, such as GM3-P15 or GS2-OF (Figure 6C,D). Both RNA1 and RNA2 of BBWV2 were identified in four samples from GM and GS, respectively, and the proportion of RNA2 was much higher than that of RNA1 in all four samples. 

### 2.6. Assembly and Mapping of SMV-Associated Contigs

**Figure 7:**
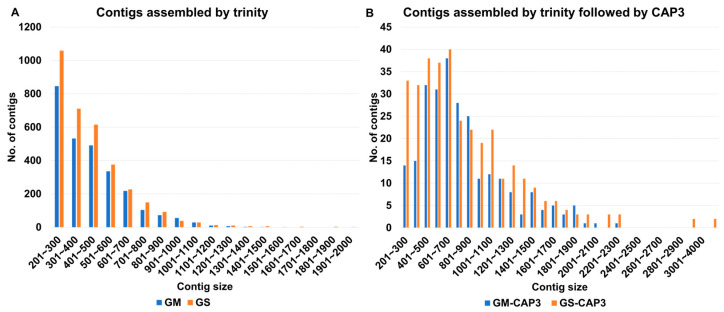
Size distribution of assembled viral contigs from GM and GS. (**A**) Viral contigs assembled using the Trinity assembler. (**B**) Viral contigs assembled using the Trinity assembler followed by the CAP3 assembler.

SMV was found to be the most abundant virus in all analyzed samples, making it natural to obtain several complete viral genome sequences, such as SMV, through RNA-sequencing, thanks to the poly(A) tail of the SMV genome. Initially, we examined the size distribution of assembled contigs from GM and GS samples, combining all viral contigs, regardless of whether they covered a complete polyprotein sequence. A total of 2706 and 3340 viral contigs were obtained from GM and GS, respectively. Most of the assembled viral contigs ranged in size from 201 bp to 300 bp, followed by 301 bp to 400 bp (Figure 7A). The longest viral contig obtained from GM was 1434 bp, while the longest from GS was 1957 bp (Figure 7A). It appears that none of the SMV genome was assembled de novo.

Subsequently, we performed assembly using the CAP3 program, combining all viral contigs from GM and GS. As a result, 256 and 344 viral contigs were obtained from GM and GS, respectively. The size of the assembled contigs was significantly longer than before. For example, the largest number of viral contigs from both GM and GS belonged to the 601 bp to 700 bp range (Figure 7B). The longest viral contig obtained from GM was 2234 bp, while the longest viral contig from GS was 4436 bp.

**Figure 8:**
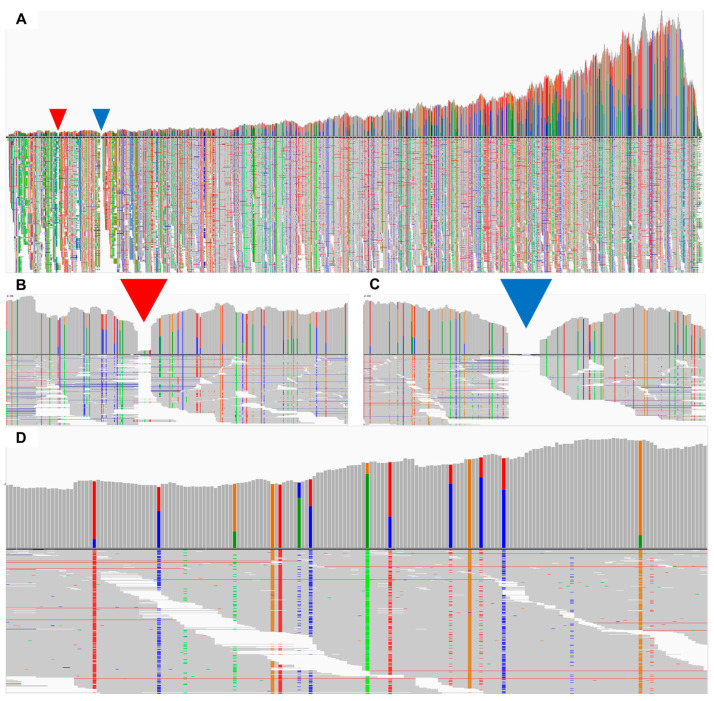
Mapping results of SMV-associated reads from the GS3-AB sample to the SMV reference genome. (**A**) Alignment outcomes of raw data sourced from the GS3-AB sample (SRR7538229) were computed against the SMV reference genome utilizing BWA software and subsequently visualized using the IGV viewer. Within the visualization, inverted triangles colored in red and blue delineate gap regions where the reads failed to align. A focused view on the mapping results in proximity to nucleotide positions 720 (**B**) and 1300 (**C**) within the SMV reference genome is presented. (**D**) Zoomed-in depiction of the mapped reads, represented by bars of distinct colors. Notably, gray signifies nucleotides not found within the reference genome, indicating insertion or deletion events (indels) relative to the reference sequence. Adenine (Green), thymine (Red), cytosine (Blue), and guanine (Orange) are color-coded representations of nucleotide bases.

To elucidate the underlying causes of the absence of fully or nearly complete de novo assembled genome sequences in this investigation, the raw data underwent alignment to the SMV reference genome utilizing the BWA aligner, followed by visualization through the IGV viewer (Figure 8). Owing to the presence of oligo-d(T) poly(A) tails, a significant proportion of reads were successfully mapped to the 3′ region of the SMV genome, with the count of mapped reads gradually diminishing toward the 5′ region of the SMV genome (Figure 8A). Furthermore, distinct gaps were identified in the 5′ regions, where reads aligned accurately with the SMV reference genomes, revealing evident breakpoints in the de novo assembly process (Figure 8B,C). Notably, the alignment of reads exposed mutations across various segments of the genome (Figure 8D). 

**Figure 9:**
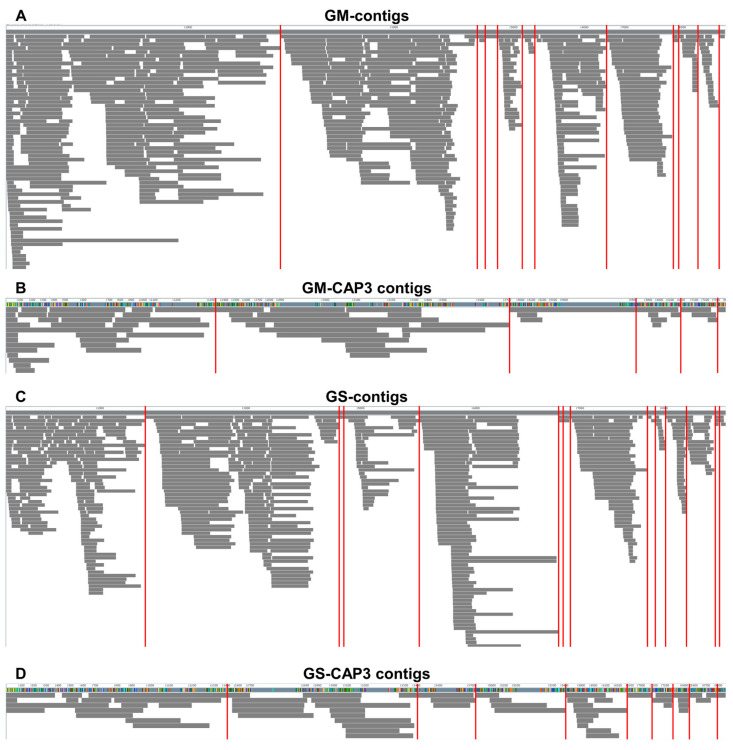
Visualization of contigs associated with SMV. (**A**) Contigs assembled from GM samples using the Trinity assembler aligned to the SMV reference genome. (**B**) Contigs assembled from GM samples using the Trinity assembler, followed by the CAP3 assembler, aligned to the SMV reference genome. (**C**) Contigs assembled from GS samples using the Trinity assembler aligned to the SMV reference genome. (**D**) Contigs assembled from GS samples using the Trinity assembler, followed by the CAP3 assembler, aligned to the SMV reference genome. The obtained viral contigs do not map to the nucleotide regions indicated by the red-colored bars on the SMV reference genome.

Moving on, we mapped the obtained contigs onto the SMV reference genome to identify discontinuous regions, represented by gaps between viral contigs associated with SMV (Figure 9). When mapping the viral contigs from GM, assembled by Trinity, we identified 11 gaps (Figure 9A). By utilizing viral contigs assembled by Trinity followed by CAP3 assemblers, the number of gaps was reduced to five (Figure 9B). Similarly, we identified 13 gaps when mapping the viral contigs obtained from GS using Trinity (Figure 9C). When mapping viral contigs from GS using Trinity and CAP3 assemblers, we identified nine gaps. Interestingly, the gaps frequently appeared at the 3′ region of the SMV genomes. As a result, we were unable to obtain any fully assembled viral genome sequence for SMV.

In addition, a substantial number of viral contigs linked to CMV were acquired (Table S6), as well as those related to BBWV2 (Table S7). In line with this, complete or near-complete genomes for CMV and BBWV2 were not successfully obtained. It is worth noting that the genomes of CMV and BBWV2 consisted of multiple viral RNA segments. However, in numerous instances, certain RNA fragment sequences were not retrieved from the corresponding samples, indicating the potential degradation of these viral RNA molecules.

## 3. Discussion

The analysis of 40 soybean transcriptome datasets allowed us to identify several viruses and examine their abundance in different samples and developmental stages. We utilized two approaches to assess viral abundance: viral contigs and viral reads. The analysis of viral contigs provided information about the number and distribution of contigs assigned to each virus. Several bioinformatic pipelines have been developed to identify viruses by high-throughput sequencing (HTS) [19,20,21]. In principle, de novo assembly followed by a BLAST search is crucial for virus identification [22]. As the size of the viral contig increases, the reliability of virus identification also increases. Additionally, the database utilized for the BLAST search should be a non-redundant protein database that encompasses all available protein sequences from known organisms. Relying on a virus-specific database may result in the misidentification of virus-like sequences from other organisms [23].

Based on the analysis of viral contigs, we identified six different viruses: BBWV2, BrYV, BWYV, CMV, MDV, and SMV. Among these, SMV was the most prevalent virus in both the *Glycine max* (GM) and *Glycine soja* (GS) cultivars, with a higher number of contigs detected in GS. As previously reported, SMV was identified as the predominant virus that infects *Glycine* species [3,5,6]. Furthermore, it is well-established that SMV can be transmitted through seeds [22]. The substantial infection rates of SMV in both wild and cultivated soybeans strongly suggest that seed transmission plays a significant role in the spread of the virus [24]. 

CMV was the second-most-abundant virus in GS, but less prevalent in GM. BBWV2 was the third-most-abundant virus in GM. Infection of soybean by CMV has been previously reported [3,25]. Moreover, previous reports indicate the presence of BWYV and MDV infections in soybean [26,27]. However, the infection of other known viruses, such as BBWV2, BrYV, and CNSV, in soybean has not been documented. Although BBWV2 and BrYV have been found to infect legume plants, like milk vetch, pea, and broad bean [26], their potential to infect soybean requires further confirmation. Notably, only a small partial sequence of CNSV was identified in this study, making it inconclusive as to whether soybean is indeed infected by CNSV. Consequently, we are able to report potential infections of soybean plants by several viruses.

To more accurately assess viral abundance, we analyzed viral reads. Among the analyzed cultivars (GM and GS), SMV was the most abundant virus based on viral reads, followed by BBWV2 and CMV. Although other viruses were found to be coinfected with SMV in soybean plants, their proportions were relatively low. The distribution of viral reads across the samples confirmed the dominance of SMV in both GM and GS, with a few samples showing higher proportions of other viruses. This finding suggests that SMV may be the primary virus responsible for viral diseases in soybean plants. Notably, open flower tissue exhibited the highest proportion of viral reads, followed by the flower bud tissue. This result indicates that viruses replicate more actively in open flowers and flower buds compared with apical buds and developing pod tissues in soybean plants. Interestingly, the apical bud stage in GM and the P15 stage in GS exhibited significant differences in viral load for SMV among different cultivars. This observation suggests that the genetic background of the cultivars may influence the replication rate of SMV in different tissues.

Based on our findings, soybean flower tissues show promise as an alternative to soybean leaves for virus detection, primarily due to their higher abundance of viral reads. Additionally, in this study, MDV was identified in two flower tissues: open flowers and flower buds of cultivated soybean plants. From these two flower tissues, we obtained six DNA fragments and a satellite DNA sequence specific to MDV. Interestingly, MDV was also detected in lily flower tissues using HTS [27]. These results suggest that flower tissues may be favorable for MDV detection and could serve as preferred samples for further studies.

The assembled viral contigs do not provide information on viral abundance. However, by analyzing viral reads and considering the number of reads mapped to the viral genome, we were able to accurately estimate the abundance of viruses. It is important to note that viral abundance based on viral reads can be influenced by the total sequence depth obtained through HTS. Therefore, proper normalization is necessary to compare viral abundance across different samples. In this study, we calculated the FPKM values to compare viral abundance between GM and GS. Interestingly, the results based on the FPKM values revealed similar viral abundances for SMV in both cultivars, while BBWV2 and CMV exhibited higher abundance in GS compared with GM. However, similar to the findings based on viral reads, the proportions of other viruses remained relatively low when considering FPKM values. Overall, our study demonstrates the utility of FPKM values in estimating viral abundance using HTS.

The comparison of viral abundance across developmental stages revealed no significant differences in viral abundance among the stages in both GM and GS. However, an intriguing finding emerged when comparing different GM cultivars at the AB stage. Specifically, GM1 and GM4 exhibited higher viral abundance compared with GM2 and GM3, with this difference being statistically significant. These results suggest that viral abundance can vary depending on the soybean cultivar, potentially due to genetic factors such as virus resistance [28]. 

In the study, the proportions of individual segments of the MDV virus were analyzed. The MDV R segment was found to have the highest proportion in both the GM3-OF and GM4-FB samples. This analysis provided insights into the composition and distribution of MDV in the samples. Interestingly, we were able to obtain circular DNA genome sequences of MDV through RNA sequencing using libraries prepared with oligo(dT) to capture mRNAs with poly(A) tails. It is speculated that the oligo(dT) might bind to the A-rich regions in the MDV genome.

The presence of DNA viral sequences in RNA-sequencing data can be attributed to various factors [29]. Firstly, DNA viruses with circular genomes can undergo transcription, resulting in the generation of viral RNA molecules that can be captured during mRNA enrichment. Secondly, transcripts of viral genes, despite the viral genome being composed of DNA, can be transcribed into RNA molecules and sequenced. Additionally, RNA intermediates produced during the replication process of DNA viruses can be targeted and captured during RNA-sequencing library preparation. Finally, host–virus interactions during the viral replication cycle can lead to the generation of chimeric transcripts containing both viral and host RNA sequences, which can be sequenced. However, it is crucial to consider the possibility of contamination or misannotation when detecting DNA viral sequences in RNA-sequencing data. Further validation and analysis, such as confirming the presence of viral DNA using PCR or sequencing specific viral regions, are typically required to ensure the accuracy of detecting DNA viruses in RNA-sequencing datasets [30].

Regrettably, despite our efforts, complete genome sequences for several identified viruses, including SMV, CMV, and BBWV2, could not be obtained through RNA sequencing. We meticulously reviewed our data analysis procedures, including the parameters for de novo transcriptome assembly, to rule out potential issues. In this pursuit, we replicated the analyses performed in our prior studies, which successfully generated complete viral genomes for SMV, CMV, and BBWV2 [3,5,31]. The outcome of this comparative analysis affirmed the integrity of our data analysis process.

Our findings led us to speculate on a range of potential reasons for the challenges encountered. Firstly, we contemplated the possibility of RNA degradation within the samples utilized for RNA library construction. RNA degradation can result in a reduction in read lengths relative to intact RNA, uneven coverage across transcripts with an overrepresentation of the 3′ ends, diminished coverage depth, biased poly(A) coverage toward the 3′ end, and an escalation in duplication rates. Although SMV is characterized by a poly(A) tail that facilitates its detection in RNA sequencing, our de novo assembly and read mapping efforts disclosed numerous gap regions that remained unmapped by the reads. This recurrent pattern hindered the acquisition of complete genome sequences, particularly for the target virus with a higher read count. Additionally, viruses harboring multiple RNA segments, like CMV and BBWV2, yielded only partial sequences from a single sample due to the potential degradation of certain RNA segments. These circumstances underscore the complexities inherent in such multipartite genomes. The second factor contributing to our challenges was the presence of diverse virus variants, exemplified by SMV, within the same sequencing library. The complexity of SMV quasispecies, referring to closely related viral variants, complicates assembling complete genomes using RNA sequencing alone [32]. Gaps in mapping SMV contigs indicate incomplete or fragmented sequences, possibly due to co-infection with different SMV variants. Particularly, the 3′ regions of SMV genomes display greater genetic divergence, hindering complete genome assembly with RNA-sequencing data. The concurrent infection of these variants creates gaps during the alignment of SMV contigs to the reference genome, particularly in the 3′ regions, highlighting significant divergence complexity. Lastly, we contemplated the influence of total RNA extraction from non-leaf samples, such as apical buds, flower buds, flowers, and pods. These materials pose inherent difficulties in achieving pristine RNA extraction, which could introduce artifacts into the sequencing data. 

In conclusion, despite encountering these obstacles, our study contributes valuable insights into the intricacies of viral genome sequencing, particularly in the context of complex multipartite genomes and co-infections. Our efforts shed light on the importance of considering factors such as RNA degradation, variant co-infections, and challenging source materials when pursuing comprehensive genome sequences through RNA-sequencing techniques. 

Overall, our analysis provides a comprehensive understanding of viral identification and abundance in soybean transcriptome datasets. The combination of viral contigs, viral reads, and FPKM values allowed us to assess viral abundance from different perspectives. The results highlight the dominance of SMV and the variations in viral abundance among different samples and developmental stages. These findings contribute to our knowledge of viral infections in soybean plants and can be valuable for further studies on virus–host interactions and crop-protection strategies.

## 4. Materials and Methods

### 4.1. Sample Collection and RNA-Sequencing

The samples used in this study were previously described [17]. In summary, four different varieties of *Glycine max* (GM) and *Glycine soja* (GS), with diverse flower and seed coat colors, were selected for transcriptome analysis. RNA sequencing was performed on samples obtained at five specific developmental stages: apical buds (ABs), flower buds (FBs), open flowers (OFs), and developing pods at five (P05) and 15 days (P15) after fertilization. It is worth noting that all soybean plants were cultivated under natural conditions in May 2014 at an experimental field located at the Institute of Botany in Beijing, China.

Upon collection, the samples were immediately frozen in liquid nitrogen and stored at −70 °C. Total RNA extraction was carried out using the SV Total RNA Isolation System (Promega, Madison, WI, USA), which employs a spin column-based purification method to effectively eliminate contaminants and obtain pure RNA suitable for downstream applications. The sequencing of 40 libraries was performed using the Illumina HiSeq4000 system with paired-end reads of 100 bp each.

### 4.2. Processing of Raw Data and De Novo Assembly of the Transcriptome

Raw data sets associated with the accession number PRJNA481793 (SRR7538190-SRR7538229) were obtained from the National Center for Biotechnology Information (NCBI) Sequence Read Archive (SRA) database. To convert the downloaded SRA data into FASTQ format, the SRA-Toolkit version v3.0.7 (https://hpc.nih.gov/apps/sratoolkit.html) was utilized (accessed on 1 March 2023). Following that, the raw FASTQ files underwent quality control, which involved trimming and filtering low-quality reads. This step was performed using the BBDuk program version v39.01 (https://jgi.doe.gov/data-and-tools/software-tools/bbtools/bb-tools-user-guide/bbduk-guide/) (accessed on 1 March 2023). The resulting high-quality reads were then subjected to de novo transcriptome assembly using Trinity version 2.15.0 with default parameters (accessed on 1 March 2023).

### 4.3. Identification of Viral Contigs through BLASTX Search

The assembled contigs from each library were subjected to a BLASTX search against the viral protein database obtained from NCBI (https://www.ncbi.nlm.nih.gov/genome/viruses/) using an E-value cutoff of 1 × 10^−10^ (accessed on 1 March 2023). Contigs showing sequence similarity to viral proteins were further analyzed through a BLASTX search against the non-redundant protein database at NCBI to specifically identify viral contigs. The identified viral contigs were then categorized based on their corresponding virus species. To align the raw sequence reads with the reference viral genomes, BWA aligner version 0.7.17 was employed with default parameters (accessed on 1 March 2023) [33]. The coverage, viral reads, and fragments per kilobase of transcript per million (FPKM) for each virus in individual samples were calculated using the eXpress program version v1.5.1 (https://pachterlab.github.io/eXpress/manual.html) (accessed on 1 March 2023) based on the SAM file.

In this study, the virus identification threshold was determined using the following parameters. Firstly, the presence of a virus-associated contig with a size greater than 200 bp was required for identification. Secondly, a minimum of 50 virus-associated reads needed to be detected. Thirdly, the identification of virus-associated contigs for the target virus was required in at least two different libraries. Fourthly, previous reports of the target virus infecting soybean plants were considered as confirmation. If an insufficient number of virus-associated contigs, viral reads, or instances of the target virus infection were found, further investigation across various samples was conducted. Applying these criteria, we successfully identified six distinct viruses: BBWV2, BrYV, BWYV, CMV, MDV, and SMV.

### 4.4. Annotation of the Viral Contigs

The viral contigs were subjected to open reading frame (ORF) prediction using the ORFfinder program available at NCBI (https://www.ncbi.nlm.nih.gov/orffinder/) (accessed on 1 March 2023). To ensure accurate analysis, reverse sequences were converted using the DNA Reverse Complement Calculator (https://jamiemcgowan.ie/bioinf/complement.html) (accessed on 1 March 2023). The predicted ORFs were then analyzed through a BLASTX search against the non-redundant protein database to identify viral genomic features. This comparison with previously known viral genomes enabled the identification of conserved motifs and potential functional elements present in the viral contigs.

### 4.5. Assembly of Contigs and Mapping of Viral Contigs on the Reference Viral Genome

The viral contigs identified through the BLASTX search were subjected to another round of assembly using the CAP3 assembler version 10.2011 with default parameters (accessed on 1 March 2023) [30]. Subsequently, the assembled contigs were subjected to a BLASTX search against the non-redundant protein database using diamond version 2.1.8 with an output option for daa format (accessed on 1 March 2023) [34]. The resulting daa files were then visualized using MEGAN6 version 6.24.20 (accessed on 1 March 2023) to display the mapping results of the contigs onto the reference genomes of SMV [35].

## 5. Conclusions

In this study, the analysis of 40 soybean transcriptome datasets led to the identification of six different viruses: BBWV2, BrYV, BWYV, CMV, MDV, and SMV. Among these, SMV was found to be the most prevalent virus in both the *Glycine max* (GM) and *Glycine soja* (GS) cultivars. The distribution of viral contigs varied across different samples and developmental stages, with flower buds (FBs) exhibiting a higher number of viral contigs in GM, while FB and open flowers (OFs) had higher viral contig numbers in GS. Overall, FB showed the highest number of viral contigs in both GM and GS. The analysis of viral reads indicated that SMV was the most abundant virus in both GM and GS, although BBWV2 and CMV had higher abundance in GS compared with GM. Moreover, viral proportions varied across developmental stages, with open flowers showing the highest proportion of viral reads. When comparing viral abundance based on viral reads and the FPKM values, SMV abundance appeared similar between GM and GS based on the FPKM values, while the proportions of BBWV2 and CMV were higher in GS based on the FPKM values compared with viral reads. There were no significant differences in viral abundance between GM and GS at the five different developmental stages based on FPKM values, except for a significant difference observed among the four different GM cultivars at the apical buds (ABs) stage. Furthermore, the presence of MDV composed of multiple segments was identified in two samples from GM, and the proportions of individual MDV segments varied within the samples. Overall, this study provides valuable insights into the prevalence, distribution, and abundance of various viruses in soybean transcriptome datasets, shedding light on their dynamics across different cultivars and developmental stages.

## Figures and Tables

**Table 1:** Information on cultivated soybean and wild soybean used for transcriptome analysis.

Cultivar Name	Species	Abbreviations	Flower Color	Seed Coat Color
Hefeng 48	*Glycine max*	GM1	purple	yellow
Donong 53	*Glycine max*	GM2	white	yellow
Nenfeng16	*Glycine max*	GM3	white	yellow
Heinong 35	*Glycine max*	GM4	white	yellow
01-737	*Glycine soja*	GS1	purple	black
200201-6	*Glycine soja*	GS2	purple	black
01-590	*Glycine soja*	GS3	purple	brown
01-555	*Glycine soja*	GS4	purple	green

## Data Availability

The raw data for this study are available from the SRA database in NCBI under the following accession numbers: SRR7538190-SRR7538229.

## Supplementary Materials

The following supporting information can be downloaded at: https://www.mdpi.com/article/10.3390/plants12183257/s1, Table S1: Summary of trimmed raw data. Table S2: Summary of virus-associated contigs per sample. Table S3: Summary of virus-associated reads per sample. Table S4: Summary of coverage for individual virus segments per sample. Table S5: Summary of FPKM values for individual virus fragments per sample. Table S6: Assembled sequences associated with CMV. Table S7: Assembled sequences associated with BBWV2.
